# Anticipating Parenthood Among Lesbian, Gay, Bisexual, and Heterosexual Young Adults Without Children in Portugal: Predictors and Profiles

**DOI:** 10.3389/fpsyg.2020.01058

**Published:** 2020-06-10

**Authors:** Jorge Gato, Daniela Leal, Susana Coimbra, Fiona Tasker

**Affiliations:** ^1^Faculty of Psychology and Education Sciences, University of Porto, Porto, Portugal; ^2^Department of Psychological Sciences, Birkbeck, University of London, London, United Kingdom

**Keywords:** parenthood intentions, lesbian women, gay men, bisexual individuals, stigma, predictors, cluster analysis

## Abstract

Parenthood is a highly valued life goal, independent of one’s sexual orientation. However, the majority of studies exploring young adults’ parenthood plans have relied exclusively on samples of heterosexual individuals. This study aimed (i) to explore differences in parenthood intentions as a function of sexual orientation, (ii) to investigate to what extent sociodemographic and psychological characteristics predict parenthood intentions of lesbian, gay, bisexual (LGB), and heterosexual individuals, (iii) to test the mediating effect of stigma between sexual orientation and parenthood intentions, and (iv) to identify and characterize profiles of prospective parenthood (through cluster analysis). Data were gathered using an online survey from 375 self-identified LGB and heterosexual young adults without children in Portugal, with a mean age of 25.83 years old (*SD* = 4.49). Findings indicated that LGB individuals were less likely to intend to have children than heterosexual individuals; furthermore, among LGB individuals, lesbian women expressed stronger intentions to have children than did gay men. Similarities between heterosexual and LGB young adults were observed concerning the psychological determinants of parenthood intentions. Four distinctive profiles of prospective parenthood were identified: aspiring parents not anticipating stigma, aspiring parents anticipating stigma, childfree intent, and childfree ambivalent. Lesbian and bisexual women mostly populated the childfree ambivalent cluster; in contrast, the aspiring parents anticipating stigma cluster contained an overrepresentation of men, including sexual minority men. Professionals may want to attend to communalities and specificities of prospective parenthood as a function of sexual orientation, in order to provide unbiased and culturally competent support to sexual minority individuals.

## Introduction

The process of family formation usually begins during young adulthood (McGoldrick et al., 2015). In the few last decades, the process of transition to adulthood has undergone profound changes in Portugal, converging with that of other western societies: concomitant with a longer educational pathway into adulthood there have been delays in the assumption of both conjugal and parental roles (Oliveira et al., 2014; PORDATA,2019a). In spite of these changes, parenthood is still a highly desired and anticipated life goal, seen by many people as an important developmental milestone in their adult life course, independent of their sexual orientation (Goldberg et al., 2012; Gato et al., 2017).

Parenthood aspirations have been operationalized in various ways, such as desires, intentions, likelihood estimations, attitudes toward childlessness, or even a parenting continuum (for a review see Gato et al., 2017). Parenting desires correspond to the extent to which one wishes or wants to have children, whereas intentions are related to decisions or plans concerning parenthood (Riskind and Patterson, 2010). Intentions are usually a consequence of the deliberation of wishes and desires and mark the transition to the pre-action phase (Baiocco and Laghi, 2013).

Most studies exploring young adults’ parenthood plans have relied exclusively on samples of heterosexual individuals (Cohler and Michaels, 2013). In fact, given prevailing societal prejudice and discrimination against sexual minority individuals, the interest in the parenthood plans of lesbian, gay, and bisexual (LGB) individuals is quite recent. Sexual minority persons face many barriers when they envisage parenthood (Gato et al., 2017) and that may explain why they express fewer desires, intentions, and expectations of having children than do heterosexual persons (e.g., Patterson and Riskind, 2010; Riskind and Patterson, 2010; Goldberg et al., 2012; Shenkman, 2012; Baiocco and Laghi, 2013; Riskind et al., 2013; Riskind and Tornello, 2017; Simon et al., 2018; Gato et al., 2019; Leal et al., 2019b; Salinas-Quiroz et al., 2019; Tate and Patterson, 2019a, b). Parenthood among bisexual individuals is also relatively understudied. In one U.S. study, bisexual individuals’ parenthood intentions generally seemed to be closer to those of heterosexual individuals than to the ones of lesbian women and gay men (Riskind and Tornello, 2017). However, in a previous study conducted in Portugal, no differences were found between lesbian and bisexual women’s parenthood intentions (Gato et al., 2019). Furthermore, studies have suggested that bisexual women who are partnered with women in fact have similar desires and intentions to those of lesbian women (Ross et al., 2012; Delvoye and Tasker, 2016; Riskind and Tornello, 2017).

One of the barriers to the parenthood aspirations of LGB individuals relates to the experience or anticipation of stigma upon parenthood (Gartrell et al., 2005; Bos and van Balen, 2008; Eady et al., 2009; Riskind et al., 2013; Bauermeister, 2014; Gato et al., 2017, 2019; Scandurra et al., 2019; Simon et al., 2019). Institutional heterosexism can be observed in many legislatures which explicitly prohibit adoption by sexual minority individuals or same-sex couples and/or obstruct these individuals’ access to Assisted Reproductive Technology (ART) services (Gato et al., 2017). For instance, the lesbian and gay participants in Riskind et al.’s (2013) study who were generally living in more favorable social climates in the United States regarding the rights of sexual minorities were also more likely to express confidence that they could become parents in the future. Bauermeister (2014) also verified that the existence of legal restrictions (e.g., same-sex marriage, adoption, etc.) moderated the relation between the fatherhood aspirations of gay men and their psychological well-being. Fatherhood aspirations were associated with fewer depressive symptoms and higher self-esteem scores among participants living in U.S. states without discriminatory policies, whereas the opposite was true in states with discriminatory policies. Besides discriminatory laws, gate-keeping processes and the personal biases of professionals working in adoption agencies, reproductive health services, or in human services in general may also hinder the progression of LGB individuals’ future parenthood projects (e.g., Hicks, 2000; Matthews and Cramer, 2006; Yager et al., 2010; Mellish et al., 2013; Kimberly and Moore, 2015; Tasker and Bellamy, 2019). Among the LGBT community (lesbian, gay, bisexual, and transgender individuals), parenthood might be considered as “heteronormative” and, thus, result in the exclusion of LGBT parents in this community (Simon et al., 2019). In this regard, Salinas-Quiroz et al. (2019) conceptualized the “homonormative family model” which includes a same-sex monogamous couple with children.

In Portugal, bills in favor of LGB individuals’ parental rights are very recent: adoption by same-sex couples and public funded access to ART for all women, irrespective of their sexual orientation, relational status, and infertility status were only approved in 2016. Furthermore, moderate to high levels of prejudice against LGB persons have been noted in this country (FRA, 2014; Eurobarometer, 2019).

Investigating attitudes toward same-sex adoptive families among Portuguese students from the helping professions, Gato and Fontaine (2016, 2017) found an association between heterosexism and negative attitudes toward adoption by lesbian women and gay men. Also in Portugal, Xavier et al. (2017) identified continuing reservations concerning same-sex couples’ access to parenthood, particularly among lawyers/attorneys with experience in the area of family and parenting. More recently, Gato et al. (submitted) aimed to understand how Portuguese adoption professionals conceptualized and prepared to work with LGB parents and verified that the discourses of these professionals oscillated between awareness of the existing stigma in Portugal against sexual minorities and heteronormative stances regarding same-sex couple adoption. Thus, there are reasons to believe that both experienced and anticipated stigma may interfere with sexual minority persons’ parental decisions.

Our knowledge of the factors shaping parenthood intentions of LGB individuals is still scarce (e.g., Baiocco and Laghi, 2013; Riskind et al., 2013; Costa and Bidell, 2017; Gato et al., 2017, 2019; Salinas-Quiroz et al., 2019; Scandurra et al., 2019; Tate et al., 2019). The current study aims to contribute to fill in this gap, by characterizing parenthood intentions amongst LGB and heterosexual young adults without children. More specifically, we aimed (i) to investigate differences in parenthood intentions as function of sexual orientation, (ii) to explore the role of sociodemographic and psychological characteristics in parenthood intentions, (iii) to test if stigma mediated the effect of sexual orientation on parenthood intentions, and (iv) to identify profiles of prospective parenthood, by describing the characteristics of those who belong to distinct groups who differ with respect to their views on becoming a parent in the future.

### Psychological Predictors of Parenthood Aspirations

In common with any other psychological construct, parenthood aspirations depend upon many contextual and individual variables. Thus, research has considered the influence of sociodemographic and psychological factors on parenthood intentions – and, whenever applicable, on associated concepts such as desires or expectations – of both LGB and heterosexual individuals. The association between psychological variables and parenthood intentions remains relatively understudied, especially among LGB individuals (Tate et al., 2019). Psychological factors have been conceptualized in different ways, such as motivations for pursuing parenthood (Goldberg et al., 2012), attitudes toward infants, children, and parenthood (Tate and Patterson, 2019b), or perceptions of parenting (Lawson, 2004; Baiocco and Laghi, 2013; Gato et al., 2019; Leal et al., 2019b).

Different psychological approaches have been used to investigate attitudes toward parenthood. For instance, Hoffman (1987) examined the perceived value of children to prospective parents. Other perspectives sought to examine appeal of parenthood by looking at the relationship between parenting desire and psychological and demographic factors (e.g., Gerson, 1986). According to Lawson (2004), examinations of the perceptions of the parenthood experience need to go beyond both the needs that children can fulfill for adults and the intensity of the desire for a child to encompass an investigation of the complex interplay between what can be gained and what can be lost in various domains central to life satisfaction (personal, relational, etc.) through parenthood. In essence, such a perspective is organized around the central construct of the anticipated or lived experience of parenthood. In the present work, we adopt Lawson’s (2004) approach – perceptions of the parenting experience – as our psychological framework for parenthood intentions.

Perceptions of the parenting experience encompass many facets of parenthood situations that are salient to individuals’ lives, namely the perceived emotional enrichment brought by children, perceptions of continuity or generativity, commitment associated with parenthood, anticipated social support from family or the community, feelings of isolation upon parenthood, and the instrumental, emotional, and physical costs associated with having a child. Next, we will review evidence of the association between these (or similar) perceptions of the parenting experience and the parenthood intentions of LGB individuals.

#### Enrichment

Children are mainly seen as a source of personal satisfaction and a major emotional investment (Giddens, 2005). Not surprisingly, the appreciation of children as an enriching factor in one’s life is an important parental motivation factor identified both among heterosexual persons (Dion, 1995; Langridge et al., 2005; Cassidy and Sintrovani, 2008) and lesbian women and gay men (Siegenthaler and Bigner, 2000; Bos et al., 2003; Goldberg et al., 2012). Consistently, on a subscale measuring the enrichment a child would bring to the lives of their parents, Lawson (2004) found that individuals whose stated intentions were to have children had higher scores than those whose did not state an intention to have children.

Comparative studies nevertheless have revealed that sexual minority individuals without children anticipate lower levels of emotional benefits of the parent-child bond and enjoyment of children than do their heterosexual peers (Baiocco and Laghi, 2013; Leal et al., 2019b). In the same way, Tate and Patterson (2019b) verified that lesbian women reported that they had fewer favorable experiences with infants and/or children than did heterosexual women.

#### Continuity

The perception that a child can guarantee the continuity of the family line and can provide support later in life also has been described as a motivator for parenthood (Lawson, 2004; Langridge et al., 2005; Goldberg et al., 2012). Interestingly, heterosexual men in Langridge et al.’s (2005) study were more likely than women to identify “continuing the family name” as a motivator for parenthood. However, lesbian women seemed less focused on generativity and passing on of family tradition than heterosexual women (Siegenthaler and Bigner, 2000). To our knowledge, published studies have not yet examined lineage consideration as a factor for gay men or bisexual people.

#### Social Support

The availability of people within personal social networks who can offer comfort, love, and encouragement is of the utmost important for the well-being of all individuals (Sarason et al., 1983). Regarding sexual minority individuals, some studies reported that they may be disadvantaged regarding social support, especially within their families (e.g., Tate et al., 2019). Lacking this type of support, LGB persons sometimes depend upon other relational networks, such as friends or former partners (Weston, 1991; Lyons et al., 2013; Knauer, 2016; Leal et al., 2019a). Other studies suggest that after becoming parents, lesbian women and gay men report, on the one hand, enhancement of the relational bonds with their parents (DeMino et al., 2007; Bergman et al., 2010; Goldberg and Smith, 2011) and, on the other hand, an increased distance toward the LGBT community (Gabb, 2004; Mallon, 2004; Gianino, 2008; Simon et al., 2019). Somewhat of a paradox is the observation that while access to parenthood is widely regarded as a universal right among the LGBT community, becoming a parent is still often considered as a heteronormative act. In this regard, sexual minority women in Simon et al. (2019) study expected less support from friends when they had children.

Different aspects of social support have been associated with both heterosexual and LGB individuals’ parenthood intentions. Regarding the former, those who feel close to parents and other family members, who are involved in long-term romantic partnerships, and who have supportive social networks are more likely than others to report intending to become parents (Starrels and Holm, 2000; Lawson, 2004; Langridge et al., 2005). As for sexual minority individuals, the lesbian women and gay men without children in Baiocco and Laghi’s (2013) study reported being less confident about receiving social support as parents in the future than did heterosexual counterparts. According to the authors, these results seemed to reflect the social and legal climate in Italy, where negative attitudes toward lesbian and gay parenthood prevailed, and where it seemed unlikely in the near future for same-sex couples to access rights to civil partnerships and legal marriage, foster care, or adoption. Also in Italy, Scandurra et al. (2019) verified that support from family, or that of significant people, could act as a buffer against the effect of stigma on parenthood desires and intentions. Leal et al. (2019b) found that, irrespective of sexual orientation, individuals without children in Portugal anticipated more social support in parenthood and less stigma if they decided to have children, compared to their counterparts from the United Kingdom. This seemed to apply to heterosexual and to LGB persons equally, with the more familistic culture of Portugal acting as a centripetal force pulling family members together across the generations (Hofstede, 2011; McGoldrick et al., 2015; Steinbach et al., 2016; Tanaka and Johnson, 2016). Regarding the predictive power of social support aspects on parenthood intentions, Tate et al. (2019) found that having more favorable parental relationships and more close friends were associated with greater likelihood of parenthood intentions, irrespective of a participant’s sexual orientation.

#### Financial, Emotional, and Physical Costs

Most reviewed studies have shown that perceptions of costs are negatively associated with parenthood intentions. Regarding financial aspects, during the last decade high youth unemployment rates and the precariousness of existing jobs have led to financial instability and to the postponement of family projects by Portuguese young adults (Oliveira et al., 2014; PORDATA,2019a). In the case of sexual minority persons, both the stigma and costs associated with adoption and assisted reproduction (Mezey, 2008; Downing et al., 2009; Goldberg et al., 2012; Riskind et al., 2013; Blanchfield and Patterson, 2015; Simon et al., 2018; Tate et al., 2019) make entry into parenthood a more costly social and economic undertaking than for heterosexual persons (Riskind et al., 2013; Blanchfield and Patterson, 2015; Simon et al., 2018; Tate et al., 2019). In fact, Tate and Patterson (2019b) found that lesbian women perceived parenthood as having a considerable cost and that this alone largely accounted for differences in parenthood aspirations between them and their heterosexual counterparts.

Nevertheless, like other parenting perceptions (e.g., social support), differences in anticipated social and economic costs seem to be moderated by factors such as culture. Thus, Leal et al. (2019b) noted more sizeable differences between sexual minority and heterosexual persons without children in the United Kingdom than in Portugal. In the United Kingdom, LGB individuals perceived parenthood to be less of a source of psychological enrichment, anticipated greater isolation upon parenthood, and also perceived higher costs involved in parenthood compared to their heterosexual counterparts. Not so in Portugal where a Southern European culture favored a more pronatalist and familistic cultural outlook than in the United Kingdom (Hofstede, 2011; Steinbach et al., 2016; Tanaka and Johnson, 2016). Lawson (2004) found no association between the evaluation of costs associated with parenting and parenthood intentions. According to Lawson these costs may be perceived simply as an inherent part of the parenting experience by all individuals, regardless of their parenthood intentions (Lawson, 2004). But a plausible alternative is that this may be a facet of Lawson’s sample characteristics. Although recognized, the costs of parenting may not yet have been salient to the reproductive decisions of studied young adults without children, many of whom may be weighing up parenthood as a distant future possibility.

#### Commitment and Isolation

The level of commitment associated with parenting a child and the imposition of a child upon daily life are both negative perceptions of parenthood that apparently were not related to stated intent to become a parent in Lawson’s (2004) study. In addition Leal et al. (2019b) did not find any differences in these aspects between heterosexual and LGB individuals studied in the United Kingdom or Portugal.

#### Anticipation of Stigma Upon Parenthood

Although not considered in Lawson’s original framework of intent to parent, there are reasons to believe that anticipated stigma may affect decision making (Hicks, 2000; Gartrell et al., 2005; Matthews and Cramer, 2006; Bos and van Balen, 2008; Eady et al., 2009; Yager et al., 2010; Mellish et al., 2013; Riskind et al., 2013; Bauermeister, 2014; FRA, 2014; Kimberly and Moore, 2015; Gato and Fontaine, 2016, 2017; Gato et al., 2017, 2019; Xavier et al., 2017; Eurobarometer, 2019; Scandurra et al., 2019; Tasker and Bellamy, 2019). In this regard, using a general measure of anticipated stigma upon parenthood (i.e., eliciting unfavorable reactions from others as a parent), Gato et al. (2019) found that lesbian women considered themselves at a higher risk of becoming a victim of social stigma as a mother than did either bisexual or heterosexual women in Portugal. In addition, anticipated stigma upon parenthood negatively predicted women’s parenthood intentions, independently of their sexual orientation.

### Sociodemographic Predictors of Parenthood Aspirations

Sociodemographic predictors of parenthood aspirations can include factors such as gender, age, income, professional status, educational level, relationship status, and religion.

*Gender* is one of the most studied predictors of parenthood aspirations among LGB individuals (Gato et al., 2017). Some studies have shown that lesbian women and gay men differ in their parenthood intentions: lesbian women reported both greater desire for parenthood and more intent than did their male peers (Riskind and Patterson, 2010; Baiocco and Laghi, 2013). Furthermore, gay men who desired to become a parent were less likely than heterosexual men to intend to have children, whereas this discrepancy was not observed among lesbian women (Riskind and Patterson, 2010; Baiocco and Laghi, 2013). Furthermore, gender was notable as a significant predictor of parenthood aspirations among monosexual and plurisexual persons in Mexico (Salinas-Quiroz et al., 2019). A gap between desire and likelihood estimations of having children also was found among Israeli gay men (Shenkman, 2012). In contrast, two studies conducted in the Portuguese context (Costa and Bidell, 2017; Leal et al., 2019b) revealed no significant gender difference in parenthood aspirations among LGB individuals.

Several factors could contribute to gender differences in parenthood aspirations. First, being able to gestate a child would ostensibly give women more options for achieving parenthood compared with men. As women, lesbian individuals also are likely to be influenced by normative gender roles. As an expression of these traditional feminine gender roles, women tend to be perceived as more committed to family life and more “maternal” (Wall, 2007). Concurrently, independent of their sexual orientation, women are more pressured to parent than men. Second, parenthood without the presence of a different gender person is still seen as contesting the heteropatriarchal definition of masculinity (Benson et al., 2005; Hicks, 2013) and also femininity (Dalton and Bielby, 2000; Epstein, 2002; Pelka, 2009). Furthermore, gay men are perceived as not only challenging the stereotype of men within mainstream culture but also within the norms surrounding gay culture, which until recently has been free of parenthood concerns (Mallon, 2004; Schacher et al., 2005; Stacey, 2006; Salvati et al., 2019). Moreover, the inaccurate association between male homosexuality and child abuse has posed an additional challenge of suspicion directed at gay men’s parenthood aspirations (Gross, 2012). Patterson and Riskind (2010) further have suggested that a lack of familiarity with alternate paths to parenthood could be involved in the reticence of gay men compared to lesbian women.

#### Age

In the United States, younger individuals are more likely to report that they intend to become parents (Williams et al., 1999). Regarding Portugal, the situation is paradoxical. On the one hand, Portuguese individuals (irrespective of sexual orientation) seem to report high levels of parenthood desires and intentions, at least when compared to their counterparts from the United Kingdom (Leal et al., 2019b). On the other hand, Portugal presently has one of the lowest fertility indexes in Europe (PORDATA,2019a), and Portuguese women’s age at the birth of their first child has been increasing steadily in the recent years from 26.5 years in 2000 to 30.4 years in 2018 (PORDATA,2019b). Given that fertility among women is associated with age, it is expected that younger women without children would express more intention for parenthood than older women in the same circumstances.

Individual lives are shaped by the historical times and places experienced across the life course (Elder, 1998). Not surprisingly, there is a cohort effect pertaining to the parenthood aspirations of LGB individuals (Gato et al., 2017). Older sexual minority individuals appear to have been exposed to discourses that equate homosexuality with childlessness (Mallon, 2004). Younger LGB individuals without children are thus more likely to desire and intend to have children than their older peers (D’Augelli et al., 2008; Rabun and Oswald, 2009; Riskind and Patterson, 2010; Riskind et al., 2013; Costa and Bidell, 2017; Gato et al., 2019). Thus, while parenthood desires and intentions might be greater in a familistic society, such as the Portuguese one, both practical and economic complexities apparently play a role in the postponement of this project (Leal et al., 2019b).

#### Professional and Educational Status

Having a job and a source of income are usually seen as instrumental precursors to having children (Umberson et al., 2010). As mentioned before, these aspects may be particularly relevant to sexual minority individuals’ parenthood decisions, given the costs associated to adoption and assisted reproduction (Mezey, 2008; Downing et al., 2009; Goldberg et al., 2012; Riskind et al., 2013; Blanchfield and Patterson, 2015; Simon et al., 2018; Tate et al., 2019). In this regard, Simon et al. (2018) found that, compared to their heterosexual and bisexual peers, lesbian women were more likely to want a permanent professional position before having children. Educational level is usually associated with higher income earning power, and it is to be expected that individuals who reach a higher level of education would also be more proficient in attaining parenthood. In fact, Tate et al. (2019) verified that education was positively associated with the parenthood intent of individuals who were without children, irrespective of their sexual orientation.

#### Relational Status

May influence decisions about future parenthood in diverse ways. Single parents usually have lower income levels than couples and this may hinder the parenthood intentions of any single individual (Maldonado, 2017). However, sexual minority people may be less vulnerable to the heteronormative narrative of having a child inside the marriage and be more willing to consider single parenthood or create a family of choice (Riggle et al., 2008). Nevertheless other research evidence appears to be contradictory perhaps with interacting cultural manifestations. Gato et al. (2019) found that relational status predicted only Portuguese heterosexual women’s parenthood desire, with partnered heterosexual participants being more likely to want to have children than their single counterparts. Relational status was not associated with Portuguese lesbian women’s desire for parenthood nor their intent to parent. Conversely, in the United States, Tate et al. (2019) showed that having a greater expectation about relationship permanence was associated with a greater likelihood of intent to become a parent irrespective of sexual orientation.

#### Religion

Individuals that are more religious are more likely to report the intention to become a parent (Hayford and Morgan, 2008). In fact, Tate et al. (2019) verified that greater religiosity was associated with a greater likelihood of parenthood intentions, irrespective of participants’ sexual orientation. Although Portugal is usually viewed as a Catholic country, this religion no longer appears to exert a prevailing influence on social values: “In a modern way, Portugal is simultaneously a secularized, religious and catholic country” (Dix, 2010, p. 25).

### Research Aims

Taking into account the literature reviewed above, we devised the following two research questions and two hypotheses (when applicable):

Research question 1: How do parenthood intentions vary as a function of sexual orientation and gender?

H1: We expected heterosexual individuals to intend more to become parents than LGB individuals (Patterson and Riskind, 2010; Riskind and Patterson, 2010; Goldberg et al., 2012; Shenkman, 2012; Baiocco and Laghi, 2013; Riskind et al., 2013; Riskind and Tornello, 2017; Simon et al., 2018; Gato et al., 2019; Leal et al., 2019b; Tate and Patterson, 2019a, b).

Research question 2: Which demographic and psychological factors are predictive of parenthood intentions, and is the nature or strength of these predictions associated with sexual orientation?

H2: We expected anticipated stigma upon parenthood to mediate the relationship between sexual orientation and parenthood intentions, i.e., the effect of stigma will affect mostly LGB individuals’ parenthood intentions (Hicks, 2000; Gartrell et al., 2005; Matthews and Cramer, 2006; Bos and van Balen, 2008; Eady et al., 2009; Yager et al., 2010; Mellish et al., 2013; Riskind et al., 2013; Bauermeister, 2014; FRA, 2014; Kimberly and Moore, 2015; Gato and Fontaine, 2016, 2017; Gato et al., 2017, 2019, Gato et al., submitted; Xavier et al., 2017; Eurobarometer, 2019; Scandurra et al., 2019; Tasker and Bellamy, 2019).

Research question 3: Taking into account the sociodemographic and psychological characteristics of participants, what profiles of prospective parenthood can be found and how are these characterized?

## Materials and Methods

### Participants

Our convenience sample was composed of 375 young adults without children, ranging from 18 to 35 years of age (*M* = 25.8; *SD* = 4.49). Sexual orientation was assessed with a categorical measure that asked participants to identify as heterosexual, bisexual, lesbian, or gay: 44 defined themselves as lesbian women, 78 as gay men, 59 as bisexual women, 7 as bisexual men, 113 as heterosexual women, and 73 as heterosexual men. Thus, 47.3% of the participants identified themselves as LGB individuals. Concerning race/ethnicity, participants answered an open-ended question and the large majority (96.5%) considered themselves to be Caucasian/European/white, while the remaining identified as “Mixed ethnicity” or “Asian.” Regarding education level, 69.1% had completed or were completing a university degree. Most participants (61.1%) reported being in a committed relationship, with a mean duration of 41.5 months (*SD* = 37.2). Differences were observed in relationship duration as function of sexual orientation, *t*(224) = 2.54, *p* = 0.012, *d* = 0.34, with heterosexual individuals having longer relationships (*M* = 47.33; *SD* = 40.08) than LGB individuals (*M* = 34.88; *SD* = 32.58). Approximately half of the sample (47.4%) were students, 7.3% were unemployed, and the remainder had a full-time or part-time job. Sample characteristics are presented in Table 1 and grouped by sexual orientation (LGB or heterosexual). The groups, as defined by sexual orientation, did not differ in age, education level, employment status, and relational status. However, the groups did differ with respect to the importance of religion in their life, with LGB persons reporting lower levels when compared to heterosexual persons.

**TABLE 1:** Sample characteristics and differences between LGB and heterosexual individuals in sociodemographic variables.

	LGB persons (*n* = 189)		Heterosexual persons (*n* = 186)		
Variable	*M*	*SD*	*M*	*SD*	
Age	26.2	4.94	25.5	3.97	*t*(358.671) = -1.458, *p* = 0.146, *d* = 0.15
Educational level	1.68	0.47	1.70	0.46	*t*(373) = 0.342, *p* = 0.732, *d* = 0.04
Religious values	2.02	1.13	2.72	1.37	*t*(354.815) = 5.352, *p* < 0.001, *d* = 0.56

	**n**	**%**	**n**	**%**	

**Gender**					
Female	103	54.5%	113	60.8%	χ^2^(1) = 1.502, *p* = 0.251, Φ = 0.06
Male	86	45.5%	73	39.2%	
**Work status**					
Work	89	47.1%	79	43.6%	χ^2^(1) = 0.442, *p* = 0.532, Φ = 0.04
Don’t work	100	52.9%	102	56.4%	
**Relational status**					
In a relationship	107	56.6%	122	65.6%	χ^2^(1) = 3.178, *p* = 0.090, Φ = -0.09
Not in a relationship	82	43.4%	64	34.4%	

To calculate the adequacy of our sample size we used *G Power Sofware* (version 3.1) (Faul et al., 2007). A power analysis, with an alpha = 0.05 and power = 0.95, showed that the projected minimum sample size needed to detect an effect size of *f* = 0.15 is *n* = 189 (for a Linear Multiple Regression, fixed model, 13 predictors). In turn, a power analysis, with an alpha = 0.05 and power = 0.95, showed that the projected minimum sample size needed to detect an effect size of *f* = 0.15 is *n* = 178 (for a Linear Multiple Regression, fixed model, 11 predictors).

### Procedure

Data were collected on-line from April to June 2015, as part of a larger study, “Lesbian, gay, and bisexual parenthood: Psychological determinants and experiences in the social context,” and given ethical approval by the institutional review board of the host institution. At the time of data collection, Portuguese law did not allow same-sex couples to adopt and only infertile women in a different-sex relationship had access to ART.

Recruitment procedures were the same for LGB and heterosexual participants and the study was advertised in general and in LGB oriented websites and social media (e.g., Facebook). The following recruitment text was used: “To have or not to have (more) children? This is a question many people ask themselves. Would you be able to help us make a difference in awareness and understanding of what influences people’s decision to parent or, if you are already a parent, what influences your decision whether or not to have more children? To participate you must be over at least 18 years of age and we are interested in your opinion regardless of your gender, sexual identity or parental status. By clicking the following link, you will find more information about this survey which is being conducted at (host institution).” The research was conducted in three countries and given the goal of the present study our sample focused on participants without children from Portugal aged under 35 years old. The age of 30 years is usually the upper limit when studying young adulthood (e.g., Oliveira et al., 2014). In this study, we opted for 35 years old, given the following specificities of the Portuguese context. In 2019, Portugal was one of the countries with the highest average age of leaving parental home (29 years) (Eurostat, 2020). In 2018, women’s age at the birth of their first child was 30.4 years (PORDATA,2019b), and mean age on first marriage was 32.1 years for women and 33.6 years for men (PORDATA,2019c). Furthermore, a traditionally high youth unemployment rate and low social expenditure targeted at young adults (e.g., housing), allied with high familistic values have an impact on the postponement of adult roles in Portugal (Oliveira et al., 2014). Finally, taking in consideration the barriers of parenthood faced by sexual minority individuals (Gato et al., 2017), it seems reasonable to assume that the transition to parenthood in this population may happen even later when compared to heterosexual individuals.

The confidentiality and anonymity of data was guaranteed with a survey link hosted on a server of the host institution which did not allow for the identification of the IP addresses. There were no mandatory answers and an “exit” or “withdraw” button on each page permitted participants who chose to do so to withdraw from the survey at any given time. Contact details for the principal researcher were provided should participants have any concerns or questions. Informed consent was presented electronically on the first page of the survey and participants indicated that they had read and understood consent information by checking boxes at the start of the questionnaire. Completing the questionnaire took no longer than 15–20 min and participation was without monetary compensation.

### Measures

#### Sociodemographics

To examine the sociodemographic composition of our sample, we asked participants about their age, gender, sexual orientation, education level, relational status, duration of relationship, and employment status. Gender was assessed as follows: 1 = Female, 2 = Male, 3 = Transgender, 4 = Transsexual, 5 = Other (Please specify). Considering sexual orientations, participants faced the following options: 1 = Heterosexual, 2 = Lesbian woman, 3 = Gay man, 4 = Bisexual, 5 = Other (please specify). In turn, educational level was assessed considering: 1 = 4 years of school, 2 = 6 years of school, 3 = 9 years of school, 4 = 12 years of school, 5 = Graduation, 6 = Master Degree, 7 = PhD. Importance of religious values was assessed using a Likert-type scale ranging from 1 (*Not important at all*) to 6 (*Extremely important*). Participants reported themselves to be in a committed relationship with a *yes* or *no* answer and the duration of the relationship was reported in months. Lastly, employment status was assessed through: 1 = Full-time job, 2 = Part-time job, 3 = Unemployed, 4 = Student, 5 = Student Worker.

#### Parenthood Intentions

To assess this variable we relied on the work of Riskind and Patterson (2010), who used a single item from the 2002 USA National Survey of Family Growth. We added to the original item, two additional items. Participants read the instruction, “Sometimes what people want and what they intend are different because they are not able to do what they want. Looking to the future…,” and were confronted with the following items, (i) “…I intend to have a child at some point” (original item), (ii) “…I have already decided that I’m going to be a parent,” and (iii) “…having a child is part of my future plans.”

Response options formed a 5-point Likert type scale, from 1 (*definitely no*) to 5 (*definitely yes*). The adaptation of the original items to the Portuguese language included a process of translation/retroversion. Subsequently, the facial validity of this version was ensured based on a cognitive interview with a group of Portuguese young adults. Small semantic adjustments to the items were made taking into account the obtained suggestions. The internal consistency value (Cronbach’s alphas) of this measure is presented in Table 2.

**TABLE 2:** Internal consistency of parenthood intentions and parenting perceptions.

	Total	Lesbian, gay, and bisexual persons	Heterosexual persons
Parenthood intentions	0.95	0.95	0.94
Enrichment	0.88	0.88	0.89
Isolation	0.76	0.75	0.76
Commitment	0.62	0.58	0.65
Continuity	0.38	0.33	0.44
Costs	0.65	0.64	0.66
Social support	0.80	0.82	0.82
Anticipation of stigma upon parenthood	0.78	0.77	0.74

#### Perceptions of Parenting

Attitudes toward parenthood were assessed using an adaptation of the Perception of Parenting Inventory (POPI; Lawson, 2004). This instrument comprises 28 items and measures dimensions of the parenting experience salient to individuals’ lives (Lawson, 2004). Considering the instructions used by Lawson (2004), participants were asked to think about what parenting a child would be like. Beyond measuring the extent to which respondents value (or disvalue) these aspects of being a parent, the instrument assesses the extent to which respondents perceive that each aspect would be (or is) personally experienced in a parenting situation. The *Enrichment* subscale was composed of eight items, and evaluates the benefits that a child would bring to the lives of their parents (e.g., “Caring for the child would bring me happiness”); *Continuity* consisted of four items assessing perceptions of generativity and continuity of the family (e.g., “The child would carry on my family line”); *Commitment* also made up of four items and tapped into the level of commitment associated with to the decision to have a child (e.g., “The child would be dependent on me for the rest of my life”); *Social support*, was composed of three items to assess the perception of social support from the family or the community (e.g., “My friends and family would help me to care for the child”). The subscale *Instrumental costs* included five items and evaluated the difficulties associated with having children (e.g., “I would worry about the child’s future”). Since this subscale included instrumental costs (e.g., financial), as well as emotional and physical costs, we decided to omit the “Instrumental” qualifier. Finally, the subscale *Isolation*, composed of four items, evaluated the interference of a child with a parent’s free time (e.g., “I would have less time to spend doing what I enjoy”). We also added five items that aimed to measure the anticipation of stigma upon parenthood: (i) “The child could be treated unfairly by people”; (ii) “My friends would find it strange if I had a child”; (iii) “Other people would find it strange if I had a child”; (iv) “People would have doubts about my parenthood skills”; and (v) “My family would find it strange if I had a child.”

Items were assessed using a Likert scale from 1 (*strongly disagree*) to 6 (*strongly agree*), with higher scores reflecting a greater endorsement that a dimension characteristic would be personally experienced. The adaptation of the instrument to the Portuguese language included a process of translation/retroversion by a qualified professional. Subsequently, the face validity of this version was ensured based on the same methodology as used with the previously described instrument. The internal consistency values (Cronbach’s alphas) of all the perceptions measured are presented in Table 2. The subscale Continuity revealed low internal consistency and was abandoned in the remainder of the present study.

### Data Analysis Procedure

To eliminate confounding effects regarding gender, age, education level, employment status, relational status, and duration of relationship from our consideration of sexual orientation on parenthood intentions, we used t-tests and chi-square tests to inspect group differences (LGB vs. heterosexual persons) regarding these variables. An independent samples *t*-test was used to explore the differences between LGB and heterosexual persons in parenthood intentions. As an exception to two-group comparisons (LGB vs. heterosexual persons), a Kruskal-Wallis test was conducted to inspect differences between lesbian women, bisexual women, gay men, bisexual men, heterosexual women, and heterosexual men, regarding parenthood intentions.

Hierarchical regression models on parenthood intentions were run separately for LGB and heterosexual participants. The first block of predictors included sociodemographic features such as gender (0 = female; 1 = male), age, educational level, work status (0 = not working; 1 = working), relational status (0 = not in a relationship 1 = in a relationship), and religiosity. The second block comprised the dimensions of parenting perceptions that correlated with parenthood intentions.

Sobel’s test is the most commonly used and recommended test to analyze the significance of simple mediation effects (Preacher, 2019). Indicators needed for Sobel’s test were calculated using SPSS and an interactive tool available online was used for the calculation of the Sobel test itself (Preacher, 2019).

To identify profiles of prospective parenthood, hierarchical cluster analysis was performed, using parenthood intentions, anticipation of stigma, and enrichment as variables. Kruskal-Wallis enabled the exploration of the different clusters. In order to further characterize the obtained clusters, associations between the different clusters and the sociodemographic characteristics (gender, sexual orientation and relational status) of the sample were explored sequentially (one demographic characteristic at a time) using the Chi-square statistic with the Monte Carlo simulation correction applied (Marôco, 2011).

## Results

We began our analyses by looking at the distribution of the continuous variables used in the study and values were within the normality range regarding both skewness (−0.390 to 1.364) and kurtosis (−0.522 to 3.99) (Table 3; Byrne, 2010; Hair et al., 2014). Next, we report results regarding: (i) differences in parenthood intentions, (ii) predictors of parenthood intentions among LGB and heterosexual individuals, (iii) mediation effects of anticipated stigma on parenthood intentions, and (iv) profiles of prospective parenthood.

**TABLE 3:** Correlations between independent variables and parenthood intentions.

Variables	*Sk*	*Ku*	*M*	*SD*	1	2	3	4	5	6	7
1. Parenthood intentions	–0.77	–0.52	3.63	1.31	−						
2. Enrichment	–1.58	4.00	5.57	1.00	0.64***	−					
3. Isolation	–0.39	0.30	4.59	1.21	−0.27***	−0.30***	−				
4. Commitment	–0.55	–0.19	5.71	0.88	0.004	0.09	0.18***	−			
5. Costs	–0.81	0.99	5.62	0.85	−0.22***	−0.23***	0.66***	0.31***	−		
6. Social support	–1.17	2.48	5.49	1.11	0.19***	0.33***	0.03	0.03	–0.02	−	
7. Anticipation of stigma upon parenthood	0.42	–0.52	3.36	1.34	−0.38***	−0.21***	0.27***	0.16**	0.27***	−0.23***	–

**p < 0.01; ***p < 0.001.

### Parenthood Intentions as a Function of Sexual Orientation and Gender

Considering the effect of sexual orientation on parenthood intentions, groups differed significantly, *t*(358.8) = 5.38, *p* < 0.001, *d* = 0.56, with LGB persons reporting lower levels of parenthood intentions (*M* = 3.47; *SD* = 1.32) when compared to their heterosexual counterparts (*M* = 4.13; *SD* = 1.06). In terms of background variables, participants differed only regarding religious values (Table 1). When we controlled for the effect of the importance of religious values on parenthood intentions, no interaction effects between sexual orientation and religious values were found, *F*(5, 345) = 0.573, *p* = 0.721, η^2^ = 0.008. Hypothesis 1 was thus confirmed.

**FIGURE 1:**
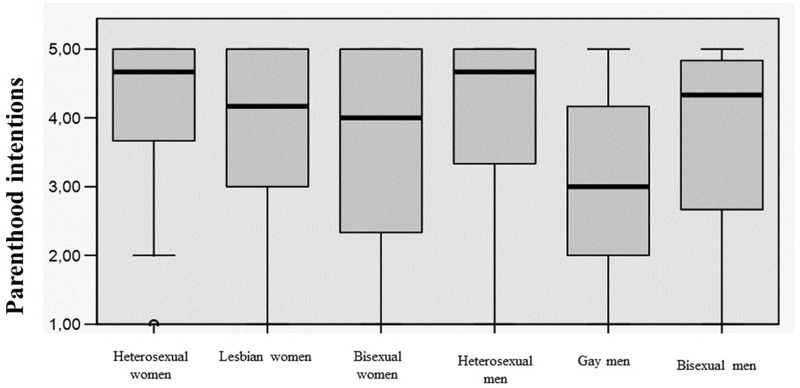
Box plot of the distribution of parenthood intentions among heterosexual women, lesbian women, bisexual women, heterosexual men, gay men, and bisexual men.

To further inspect differences in parenthood intentions as function of all groups considered (lesbian women, bisexual women, gay men, bisexual men, heterosexual women, and heterosexual men), and given imbalances in the number of participants in each group, we employed the Kruskal-Wallis non-parametric test to evaluate differences among the six groups on median change in parenthood intentions. The test, which was corrected for tied ranks, was significant and parenthood intentions were thus significantly associated with sexual orientation and gender, χ^2^(5, *N* = 375) = 37.8, *p* ≤ 0.001, η^2^ = 0.23. Pairwise comparisons revealed differences between (i) lesbian women and gay men, (ii) gay men and heterosexual men, and (iii) gay men and heterosexual women (a Bonferroni correction was applied controlling for Type I error across tests). Bisexual individuals were not significantly different from any of the other groups. When compared to gay men, lesbian women were more likely to express the intention to have children. In turn, gay men showed lower levels of parenthood intention when compared to heterosexual individuals (Figure 1).

### Predictors of Parenthood Intentions

We first examined the significant bivariate correlations between perceptions of parenting and parenthood intentions in the entire sample (see Table 3). All parenting perceptions significantly correlated with parenthood intentions, except for Commitment. We excluded this variable from further analyses in the interest of parsimony and to maximize statistical power.

We then conducted a hierarchical regression analysis with two steps: (a) sociodemographic variables and (b) parenting perceptions. We used Tolerance and VIF as multicollinearity indexes; the most common cutoff employed is a tolerance value > 0.10 corresponding to a VIF < 10. In order to assume the absence of multicollinearity, it is also important that correlations among independent variables are below.70 and/or below the correlation between each independent variable and the dependent variable (Hair et al., 2014). All indicators in our regression analyses yielded results within the established cutoff values for multicollinearity (*r* ≤ 0.66, *p* < 0.001; LGB individuals’ subsample: tolerance > 0.44; VIF < 2.26; heterosexual individuals’ subsample: tolerance > 0.48, VIF < 2.01). Regression models regarding parenthood intentions among LGB and heterosexual participants were significant, explaining respectively 48 and 46% of the outcome variable (Table 4). Concerning sociodemographic features, gender was the only significant and weak predictor of LGB participants’ parenthood intentions, suggesting that being a lesbian or bisexual woman was a predictor of planning to parent, among non-heterosexual participants. Among heterosexual participants, work and relational status were also both significant and weak predictors of parenthood intentions, such that being in a relationship and unemployed increased the likelihood of wanting to become a parent. Regarding the second block of predictors, a similar pattern of significant predictors was observed for both LGB and heterosexual samples: enrichment was a positive and moderate predictor and anticipation of stigma was a negative and weak predictor of parental intentions.

**TABLE 4:** Summary of hierarchical regression analysis for variables predicting parenthood intentions among LGB and heterosexual individuals.

	LGB persons (*n* = 158)								Heterosexual persons (*n* = 175)							
Variable	*R*^2^	Δ*R*^2^	*B*	*SE B*	95% CI	β	*t*	*P*	*R*^2^	Δ*R*^2^	*B*	*SE B*	95% CI	β	*t*	*p*
**Step 1**	0.04	0.04							0.07	0.07						
Gender			–0.36	0.16	[−0.67;-0.05]	–0.14	–2.28	0.024			0.15	0.12	[−0.09;0.39]	0.07	1.23	0.220
Age			0.003	0.02	[−0.04;0.04]	0.01	0.13	0.900			0.001	0.02	[−0.04;0.04]	0.003	0.04	0.972
Educational level			0.12	0.09	[−0.05;0.30]	0.10	1.41	0.160			0.09	0.08	[−0.07;0.24]	0.07	1.08	0.281
Work status			–0.09	0.18	[−0.43;0.25]	–0.04	–0.52	0.605			–0.34	0.15	[−0.63;-0.06]	–0.16	–2.35	0.020
Relational status			0.27	0.15	[−0.04;0.57]	0.10	1.75	0.083			0.42	0.12	[0.18;0.66]	0.19	3.42	0.001
Religiosity			–0.14	0.07	[−0.27;0.001]	–0.12	–1.96	0.052			0.06	0.04	[−0.03;0.15]	0.08	1.40	0.164
**Step 2**	0.52	0.48							0.53	0.46						
Enrichment			0.84	0.09	[0.69;1.01]	0.61	9.76	<0.001			0.62	0.07	[0.48;0.76]	0.58	8.73	<0.001
Isolation			–0.14	0.09	[−0.31;0.04]	–0.13	–1.56	0.121			–0.02	0.07	[−0.16;0.11]	–0.02	–0.32	0.751
Costs			–0.04	0.13	[−0.29;0.20]	–0.03	–0.35	0.725			–0.09	0.10	[−0.28;0.11]	–0.07	–0.89	0.377
Support			–0.06	0.07	[−0.20;0.07]	–0.06	–0.90	0.372			0.04	0.06	[−0.09;0.16]	0.04	0.59	0.558
Stigma			–0.14	0.06	[−0.26;-0.02]	–0.15	–2.34	0.020			–0.16	0.06	[−0.27;-0.05]	–0.18	–2.86	0.005

Given that we had previously detected differences in relationship duration as a function of sexual orientation, we further scrutinized whether relationship duration was associated with parenthood intent in the two subsamples. No significant correlations were detected either for heterosexual participants (*r* = −0.17, *p* = 0.069), or LGB ones (*r* = −0.13, *p* = 0.183).

### Mediation Effect of Stigma in the Relationship Between Sexual Orientation and Parenthood Intentions

**FIGURE 2:**
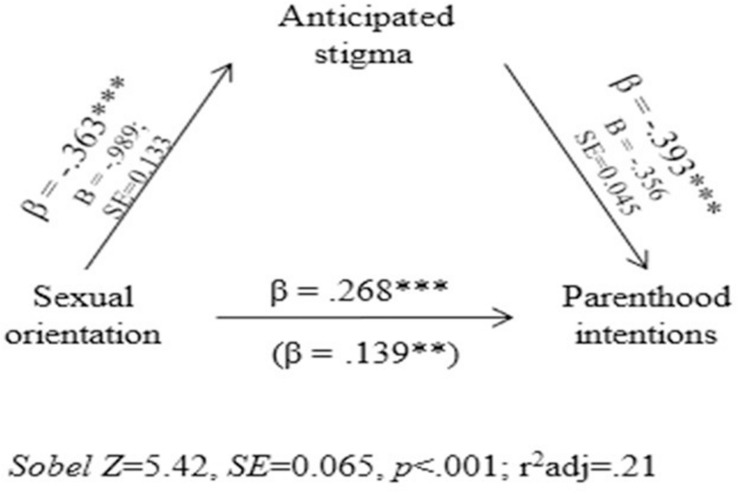
Anticipated stigma mediating the relationship between sexual orientation, and parenthood Intentions.

Anticipated stigma upon parenthood differed as a function of sexual orientation, *t*(354.12) = −7.41, *p* < 0.001, *d* = 0.77, with LGB individuals reporting higher levels (*M* = 3.87; *SD* = 1.36) than their heterosexual peers (*M* = 2.88; *SD* = 1.19). A mediation effect can occur when an independent variable affects a dependent variable through a mediating variable. As may be observed in Figure 2, anticipated stigma partially mediated the relationship between sexual orientation and parenthood intentions, as the direct effect of the sexual orientation on parenthood intentions (β = 0.268) decreased (β = 0.139) when it was mediated by anticipated stigma (48.1% of the total effect of sexual orientation on parenthood intentions was accounted for by anticipated stigma). This model explained 20% of the variance and was statistically significant (*Sobel Z* = 5.42, *SE* = 0.065, *p* < 0.001). Thus, anticipated stigma mediated parenthood intentions particularly among LGB individuals and hypothesis 2 was thus confirmed.

### Profiles of Prospective Parenthood

Given the exploratory nature of this aim, hierarchical clustering was employed. Hierarchical clustering has been the preferred approach when there are no previous hypotheses or expectations regarding the number of clusters that could be observed. Furthermore, it is also the most suitable method for a moderate sample size (under 400, not exceeding 1,000) and thus is congruent with the current study (Hair et al., 2014). Entered variables were parenthood intentions and its strongest psychological predictors (enrichment and anticipated stigma). A range between two and four clusters were requested as possible solutions, and the chosen solution followed the criteria of psychological intelligibility associated with the greatest increase of explained variance (Hair et al., 2014). Thus, the hierarchical cluster analysis revealed that the best solution for the data was a four-group clustering solution, explaining 54% of the variance (preferred over the 22.4% of two-group and the 24.8% three-group clustering solutions). These clusters were statistically distinct from each other. The results of the Kruskal-Wallis test are presented in Table 5, together with the means of the four clusters in the selected dimensions.

**TABLE 5:** Means and standard deviations of parenthood intentions, anticipation of stigma, and enrichment for each cluster.

Dimensions	Cluster 1 (*n* = 228) Aspiring parents not anticipating stigma	Cluster 2 (*n* = 7) Childfree intent	Cluster 3 (*n* = 30) Childfree ambivalent	Cluster 4 (*n* = 82) Aspiring parents anticipating stigma	χ^2^(3, *N* = 347)
**Parenthood intentions**					
*M* (*SD*)	4.32^a^ (0.83)	1.14^c^ (0.38)	2.02^c^ (0.75)	3.41^b^ (1.23)	114.986***
**Anticipation of stigma**					
*M* (*SD*)	2.61^d^ (0.85)	4.00^b,c^ (0.91)	3.69^c^ (1.02)	5.13^a,b^ (0.75)	195.816 ***
**Enrichment**					
*M* (*SD*)	5.92^a^ (0.61)	1.80^b^ 0.61	4.09^b^ (0.59)	5.75^a^ (0.71)	94.705 ***

Different letters represent statistically significant differences (***p < 0.001) and are ordered to show the increase/decrease of values. Due to an imbalance in the distribution of participants among the four clusters, we resorted to the Kruskall-Wallis test. Means and standard deviations are presented to increase readability of results, instead of ranks.

Participants in the first and largest cluster presented the highest levels of parenthood intent, the lowest levels of anticipated stigma, and were among those anticipating the highest levels of enrichment through parenthood; this cluster was named as *aspiring parents not anticipating stigma*. The *childfree intent* cluster comprised the residual number of participants who endorsed among the lowest levels of parenthood intentions and thoughts of enrichment through parenthood in the sample, alongside a close to mean level of anticipated stigma associated with parenthood. The third cluster was similar to the previous one in terms of parenthood intentions and anticipated stigma, but participants in this group presented close to mean levels of thoughts of enrichment; this cluster was named as *childfree ambivalent*. Finally, the fourth cluster, *aspiring parents anticipating stigma*, comprised participants highly motivated to become parents who anticipated both the highest levels of enrichment through parenthood but also thought they would experience high levels of stigma when performing this role.

As expected, the first cluster – the aspiring parents not anticipating stigma – was significantly populated by heterosexual women with partners. Conversely, aspiring parents anticipating stigma were mostly men and mostly non-heterosexual. The childfree ambivalent cluster was significantly associated with being a lesbian or bisexual woman. Finally, the least populated cluster – the childfree intent grouping – was mostly composed of participants who were not currently in a relationship (Table 6). Significant associations were observed between clusters and gender, χ^2^(3, *N* = 347) = 8.79, *p* = 0.032, Φ = 0.159, with women populating more the *aspiring parents not anticipating stigma* cluster and men the *aspiring parents anticipating stigma* cluster. A significant association was also found for sexual orientation, χ^2^(3, *N* = 347) = 34.0, *p* < 0.001, Φ = 0.313, with heterosexual persons over represented in the *aspiring parents not anticipating stigma* cluster and LGB persons over represented in the *aspiring parents anticipating stigma* cluster. The same was true for the interaction between gender and sexual orientation, χ^2^(9, *N* = 347) = 46.8, *p* < 0.001, Φ = 0.367, with sexual minority women predominant in the *childfree ambivalent* cluster, sexual minority men prevailing in the *aspiring parents anticipating stigma* cluster, heterosexual women predominating in the *aspiring parents not anticipating stigma* cluster, and heterosexual men underrepresented in the *aspiring parents anticipating stigma* cluster. Finally, individuals who were in a relationship were predominant in the *aspiring parents not anticipating stigma* cluster and individuals who were not in a relationship predominant in the *childfree intent* cluster, χ^2^(3, *N* = 347) = 8.04, *p* = 0.045, Φ = 0.152. No significant differences were observed regarding age χ^2^(3, *N* = 347) = 7.25, *p* = 0.064, η^2^ = 0.02; educational level, χ^2^(3, *N* = 347) = 5.29, *p* = 0.152, η^2^ = 0.02; or professional status, χ^2^(3, *N* = 342) = 4.60, *p* = 0.204, Φ = 0.116.

**TABLE 6:** Socio-demographic characteristics’ percentages in the different clusters of prospective parenthood.

	Aspiring parents not anticipating stigma (*n* = 228)	Childfree intent (*n* = 7)	Childfree ambivalent (*n* = 30)	Aspiring parents anticipating stigma (*n* = 82)	% of cases
**Gender**					
Female	63.2^1^	42.9	60	45.1^0^	58.2
Male	36.8^0^	57.1	40	54.9^1^	41.8
**Sexual orientation**					
LGB	36.4^0^	42.9	63.3	72^1^	47.3
Heterosexual	63.6^1^	57.1	36.7	28^0^	52.7
**Gender × Sexual orientation**					
LB women	22.8	14.3	43.3^1^	29.3	25.9
GB men	13.6^0^	28.6	20	42.7^1^	21.3
Heterosexual women	40.4^1^	28.6	16.7^0^	15.9^0^	32.3
Heterosexual men	23.2	28.6	20	12.2^0^	20.5
**Relational status**					
Not in a relationship	31.6^0^	71.4^1^	43.3	42.7	36
In a relationship	68.4^1^	28.6^0^	56.7	57.3	64

*0, 1 Significant association (chi-square statistics): 0 = inferior frequency of cases observed/expected; 1 = superior frequency of cases observed/expected.*

## Discussion

The main aim of this research was to characterize parenthood intentions of young adults who were without children at the time of the study, taking into account their sexual orientation. Globally, we found that LGB individuals expressed less intent to have children than did heterosexual individuals and that lesbian women were more likely to intend to have children than were gay men. Parenthood intentions of both LGB and heterosexual individuals seemed to be best predicted by similar psychological motivations, that is, by anticipating the emotional enrichment children will bring. Anticipation of stigma upon parenthood partially mediated the relationship between sexual orientation and parenthood intentions: in comparison to their heterosexual peers, LGB individuals who anticipated more stigma upon parenthood were less likely to intend to have children. In turn, four profiles of prospective parenthood were identified: aspiring parents not anticipating stigma, aspiring parents anticipating stigma, childfree intent, and childfree ambivalent. Lesbian and bisexual women were mostly represented in the childfree ambivalent cluster, while sexual minority men predominated in the aspiring parents anticipating stigma cluster.

Consistent with existent literature, LGB individuals reported lower levels of parenthood intentions than did their heterosexual counterparts (Patterson and Riskind, 2010; Riskind and Patterson, 2010; Goldberg et al., 2012; Baiocco and Laghi, 2013; Riskind and Tornello, 2017; Simon et al., 2018; Gato et al., 2019; Leal et al., 2019b; Tate and Patterson, 2019a, b). The barriers still faced by sexual minority people envisaging parenthood may be responsible for this situation (Gato et al., 2017). However, the hypothesis that sexual minority individuals may not feel as socially pressured to have children should also not be discarded as a potential explanation of these results.

In common with studies in Italy and the United States, Portuguese lesbian women in the current study reported higher levels of parenthood intent than did gay men (Riskind and Patterson, 2010; Baiocco and Laghi, 2013). However, this finding is not in accord with previous research conducted in Portugal, which was unable to detect gender differences among sexual minority individuals’ parenthood intentions (Costa and Bidell, 2017; Leal et al., 2019b). This discrepancy might stem from sample characteristics, such as age. For instance, Leal et al. (2019b) used the same instrument as we did to assess parenthood intentions, but sampled a wider age range of participants (18–45 years). As mentioned before, the absence of difference in parenting intentions as a function of gender in older sexual minority individuals might stem from a cohort effect (Elder, 1998; Mallon, 2004; Gato et al., 2017). Future research should therefore continue to investigate this issue. The fact that lesbian women reported higher levels of parenthood intent than did gay men may be attributed to the biological possibility of pregnancy and perhaps gendered views of parenting as a feminine domain (Dalton and Bielby, 2000; Epstein, 2002; Benson et al., 2005; Wall, 2007; Pelka, 2009; Hicks, 2013), prejudice against gay men as candidates for parenthood (Mallon, 2004; Schacher et al., 2005; Stacey, 2006; Berkowitz and Marsiglio, 2007; Gross, 2012), and a lack of familiarity with alternate routes to parenthood in the case of gay men (Patterson and Riskind, 2010).

Finally, bisexual individuals were not different from lesbian women or gay men, nor from heterosexual individuals regarding their parenthood intentions, a result which partially contradicts Gato et al.’s (2019) study, in which differences in parenting intentions were found between bisexual and lesbian women, and heterosexual women. However, the results from the present study are in line with those of Riskind and Tornello (2017) where differences between lesbian and bisexual women were detected. Again, these contradictory findings merit further investigation. Here particular attention should be given to the gender of the partner of bisexual individuals as perhaps being in a relationship with a different gender person might be associated with higher levels of desire for parenthood (Delvoye and Tasker, 2016; Riskind and Tornello, 2017).

The composition and strength of predictive factors for parenthood intentions were similar for both sexual orientations. Tate et al. (2019) likewise found that demographic and sociocontextual variables similarly predicted parenthood intentions among all participants, irrespective of sexual orientation. Again confirming results obtained previously, in the present study, gender predicted LGB participants’ parenthood intentions, which may be explained by the above-mentioned biological and social factors (Dalton and Bielby, 2000; Epstein, 2002; Mallon, 2004; Benson et al., 2005; Schacher et al., 2005; Stacey, 2006; Berkowitz and Marsiglio, 2007; Wall, 2007; Pelka, 2009; Patterson and Riskind, 2010; Gross, 2012; Hicks, 2013).

Notwithstanding the similarities across sexual orientation, some factors were stronger predictors for both lesbian women’s and gay men’s parenthood intentions whereas other factors were stronger for heterosexual individuals. Parenthood has historically been viewed in the context of relationships that are considered to be more permanent even if these relationships are non-marital. However, relational status predicted only the parenthood intent of heterosexual persons (Gato et al., 2019). This finding suggests that LGB persons may be more immune to heteronormative pressures to have a child within the context of marriage and more willing to create a family of choice or to have children on their own (Riggle et al., 2008). However, as Tate and Patterson (2019b) noted, although lesbian and gay people seem as likely as heterosexual individuals to desire marriage, they also seem less likely to expect that they will marry. Thus, the finding that relational status is not predictive of LGB individuals’ parenthood intent may also be interpreted as a realistic appraisal of future life circumstances.

Not having a job increased intent to become a parent among heterosexual, but not among LGB, individuals. This result apparently contradicts the fact that having a job and a source of income are usually seen as necessary instrumental conditions to have children. Participants in our study correspond to a profile of Portuguese emergent adults who are not in paid employment, yet are investing in their education, and who are probably still residing with their parents (Oliveira et al., 2014; PORDATA,2019a). A tempting explanation would be the following: because these individuals have not entered the job market yet and lack experience of personal life-family work reconciliation difficulties, they may have idealized parenthood. However, this hypothesis needs to be further explored. The fact that employment status did not seem to matter to LGB individuals’ parenthood intentions also contradicted previous results (Mezey, 2008; Downing et al., 2009; Goldberg et al., 2012; Simon et al., 2018). We wonder if this may be connected with the period of data collection, when no laws protecting parenthood among LGB individuals had yet been approved in Portugal and the actual possibility of parenthood might still have been seen as too distant.

Psychological predictors explained the major portion of the variance in parenthood intent in the current study, that is, to have children generally seemed to be more dependent upon individuals’ cognitive and emotional resources than on structural characteristics. This pattern is understandable within the modern individualization process taking place in the so-called highly industrialized societies (Beck and Beck-Gernsheim, 2002), in which emotional fulfillment and individual well-being tend to be perceived as more important determinants of individual action than do structures such as social class or kinship.

The appreciation of children as an enriching factor in one’s future life was the most significant predictor of parenthood intentions, which is consistent with the contemporary view of children as a source of personal satisfaction and a major emotional investment (Giddens, 2005). Furthermore, this pattern was independent of sexual orientation (Dion, 1995; Siegenthaler and Bigner, 2000; Bos et al., 2003; Lawson, 2004; Langridge et al., 2005; Cassidy and Sintrovani, 2008; Goldberg et al., 2012).

Similarly to Lawson’s (2004) findings, in our study it was a positive aspect of parenting (enrichment) and not negative ones (such as isolation or costs) that emerged as predictive of parenthood intentions. As Lawson stated, negative perceptions of the parenting experience, although recognized, may not yet be salient to the reproductive decisions of young adults without children. Thus, it may be the expectation of more positive aspects of parenting that distinguishes those who are motivated to be a parent from those who are not. Furthermore, in Lawson’s (2004) study, perceptions of parenting were more predictive of parenthood intentions within a general community sample than within a sample of young individuals without children, most of whom were highly educated. We concur with Lawson’s explanation for this result: attitudes, intentions, and behavioral outcomes are most strongly related when the behavior is immediate and more weakly related in the case of a potential future behavior (Ajzen and Madden, 1986). In brief, it is likely that these young people had very tentative parenting motivations at this time in their lives.

Anticipation of stigma upon parenthood mediated the relationship between sexual orientation and parenthood intentions, suggesting that this perception is indeed a deterrent to LGB individuals’ parenthood plans (Gartrell et al., 2005; Bos and van Balen, 2008; Eady et al., 2009; Gato et al., 2017, 2019; Scandurra et al., 2019). This is not surprising if we take into account the high levels of prejudice perceived by LGBT individuals in Portugal (FRA, 2014; Eurobarometer, 2019).

Nonetheless, the anticipation of stigma upon parenthood negatively predicted participants’ parenthood intentions in both LGB and heterosexual groups. In order to allow for group comparisons, the subscale “anticipated stigma upon parenthood” was composed of items that probably did not effectively capture the specificities of stigma directed at sexual minority individuals. Nevertheless, there may be various reasons for perceiving stigma upon parenthood, and these appeared to influence parenthood intent for heterosexual individuals too. Still, the fact that stigma significantly mediated the relationship between sexual orientation and parenthood intentions is indicative that this variable affects LGB individuals more than heterosexual individuals in terms of intent to parent.

The fact that heterosexual individuals mostly populated the aspiring parents not anticipating stigma group, and LGB individuals the aspiring parents anticipating stigma and childfree ambivalent groups, is illustrative of the barriers that the latter may face regarding parenthood. Clearly, parenthood is still a domain more positively considered by heterosexual individuals, in particular by heterosexual women. As we mentioned before, biological and social factors converge to possibly explain these results (Dalton and Bielby, 2000; Epstein, 2002; Mallon, 2004; Benson et al., 2005; Schacher et al., 2005; Stacey, 2006; Berkowitz and Marsiglio, 2007; Wall, 2007; Pelka, 2009; Patterson and Riskind, 2010; Gross, 2012; Hicks, 2013). When societal discrimination and stigma interfere with the aspirations of LGB individuals to have children, this might in turn have negative consequences in terms of their well-being and mental health (Hatzenbuehler et al., 2010; Shenkman, 2012; Bauermeister, 2014). Thus, the overrepresentation of LGB individuals in the childfree ambivalent and aspiring parents anticipating stigma profiles is a concerning result. More specifically, sexual minority women were overrepresented in the childfree ambivalent cluster while sexual minority men were overrepresented in the aspiring parents anticipating stigma cluster. This way, gendered views of parenting as a feminine domain (Dalton and Bielby, 2000; Epstein, 2002; Benson et al., 2005; Wall, 2007; Pelka, 2009; Hicks, 2013) as well as prejudice against gay male (prospective) parents (Mallon, 2004; Schacher et al., 2005; Stacey, 2006; Berkowitz and Marsiglio, 2007; Gross, 2012) might account for gender differences in the parenthood intent of sexual minority individuals.

### Limitations, Future Directions, and Implications for Practice

Notwithstanding its contributions, this study was not without some caveats. Our convenience sample was highly educated and thus not representative of the Portuguese population in general. In this respect, it is worth noting that while 69.1% of our participants had completed (or were completing) a university degree, in 2016 only 17.8% of the Portuguese population had attained this educational level (PORDATA, 2020). Neither age, education, nor religiosity predicted parenthood intentions in the present study and this may have been because homogeneity within the study sample restricted variation in these respects. Thus, future studies should recruit more diverse samples in terms of their social and demographic composition. Moreover, there was an imbalance within the LGB group regarding the number of bisexual men, which prevents us from drawing conclusions regarding this group. Given the nature of how the research was advertised (i.e., about attitudes to parent or not parent), the study might have drawn the attention of participants who were interested in parenthood which also imposes limitations to the generalizability of results.

The specificity of stigma directed at sexual minorities and the harmful impact it may have on parenthood intentions is an important area for future research. In particular, an examination of the mediating role of minority stress (Meyer, 2003) variables could be a fruitful research paradigm. In fact, stigma processes seem to partly explain parenthood desires and intentions of lesbian women and gay men without children (e.g., Scandurra et al., 2019).

Although previous studies have reported differences in anticipated costs, isolation, and social support as a function of participants’ sexual orientation (Baiocco and Laghi, 2013; Leal et al., 2019b; Tate et al., 2019), these were not associated with parenthood intentions in the present sample and future research should continue to investigate these factors. In the case of LGB individuals, more nuanced aspects of these motivators merit attention. Given financial costs associated with some parenthood options for LGB individuals (Riskind et al., 2013), the perception of costs associated to LGB individuals’ access to parenthood should be assessed. In the case of social support, it would be important to know specifically whether family of origin is a source of support or stigma for future family formation with children. In particular this may become more important for the present generation of young adults who are likely to be dependent upon their parents for longer compared to previous cohorts (Oliveira et al., 2014). Furthermore, young adult dependency may operate differently in familistic cultures, such as the Portuguese one (Hofstede, 2011; Steinbach et al., 2016; Tanaka and Johnson, 2016).

Our psychological predictors explained a considerable portion of the variance in both LGB and heterosexual groups. Nonetheless, other psychological variables could be evaluated in future investigations. More nuanced aspects of relationship status could also be considered in forthcoming works, such as relationship duration, expectations of relationship permanence or expectation of marriage, or whether bisexual individuals are involved in a current same-gender or different-gender partnership (Tate and Patterson, 2019a; Tate et al., 2019). Other variables that could be taken into account in future studies as predictors of parenthood intentions among LGB individuals include adherence to gender roles (Salvati et al., 2018), beliefs on children’s adjustment in same-gender parented families (Ioverno et al., 2018), or self-efficacy in the achievement of parenthood (Riskind et al., 2013; Simon et al., 2019). Furthermore, considering that sexual minority individuals may not feel as socially pressured to have children as their heterosexual peers, assessing this would also be advisable. Problems regarding the internal consistency of the subscale continuity should also be addressed.

It would be interesting to have other informants, such as partners and/or parents, to triangulate information and run interdependent analyses. These data could also enable the construction of more complex profiles of prospective parenthood (including profiling couples/families). Finally, given that the present study was conducted before the approval of laws facilitating LGB individuals’ access to parenthood, it would be interesting to investigate to what extent parenthood intentions (and their predictors) of sexual minority individuals have changed in Portugal, after these legal modifications occurred in 2016. Another important area of further study could involve the exploration of the relationship between the perceptions of parenting prior to parenthood and the actual experience of parenting both among heterosexual and LGB individuals.

Notwithstanding the limitations of our investigation, some of the strengths of this study warrant mention. First, by including both LGB and heterosexual individuals, we identified both similarities and differences in prospective parenthood that allowed for a more refined understanding of this complex process. Second, we used a multifaceted psychological framework of attitudes toward parenthood to investigate predictors and profiles of prospective parenthood. Third, we included some consideration of bisexual participants, who have often been neglected in previous prospective parenthood research. Finally, one of the major strengths of this study was the inclusion of a cluster analysis which, to our knowledge, has not been used in previous research about this topic. Cluster analysis is an exploratory analysis that tries to identify homogenous groups of cases not previously known. It is a specifically useful tool to identify different profiles resulting from the combination of different variables; these profiles include the ones that are more represented in society, but also other emergent minority profiles that also need to be considered, in both research and intervention. This assumption perfectly fitted the aims of the current study which sought to underline the heterogeneity of parenting profiles, deconstructing heteronormative stereotypes regarding parenthood.

Our results have important consequences for practice. First, there is a clear need for culturally competent professional practices that both affirm LGB individuals’ rights (American Psychological Association [APA], 2012; Moleiro et al., 2017) and consider the specificities of LGB individuals’ parenthood plans as identified in this study. Second, anti-discrimination policies protective of sexual minority persons’ parenting rights should be enacted or reinforced. Findings may be particularly important for professionals who work with sexual minority individuals in different contexts, such as schools or healthcare services (e.g., family planning consultations in primary healthcare services) (Gato et al., 2019). These professionals should be able to provide scientifically validated information about LGB parenthood and give accurate information about legal support and public services for parents and future parents.

## Conclusion

The present work has contributed to the emergent field of international research looking at the psychology of family formation in an inclusive and affirmative way. Motivations to have children are apparently similar for heterosexual and LGB individuals; nevertheless, LGB young adults reported lower levels of parenthood intent, which in turn was mediated by higher levels of anticipated stigma upon parenthood. Parenthood remains a heteronormative and feminine dominated domain, and sexual minority individuals are in a more disadvantaged position regarding parenthood aspirations.

## Data Availability Statement

The datasets for this article are not publicly available in order to protect the participants’ confidentiality. Requests for datasets should be sent to the corresponding author.

## Ethics Statement

The studies involving human participants were reviewed and approved by Comissão de Ética da Faculdade de Psicologia e de Ciências da Educação da Universidade do Porto. The patients/participants provided their written informed consent to participate in this study.

## Author Contributions

JG designed the study and took the lead in writing the manuscript. DL and SC helped with the literature review and the statistical analyses. FT collaborated in the writing of the manuscript and reviewed its contents. All authors made substantial intellectual contributions to the work, revised the manuscript, and approved it for publication.

## Conflict of Interest

The authors declare that the research was conducted in the absence of any commercial or financial relationships that could be construed as a potential conflict of interest.
